# CD3+/CD4+ cells combined with myosteatosis predict the prognosis in patients who underwent gastric cancer surgery

**DOI:** 10.1002/jcsm.13517

**Published:** 2024-06-18

**Authors:** Zhongze Du, Youming Xiao, Guiming Deng, Haibin Song, Yingwei Xue, Hongjiang Song

**Affiliations:** ^1^ Department of Gastrointestinal Surgery Harbin Medical University Cancer Hospital, Harbin Medical University Harbin Heilongjiang China; ^2^ Department of Pediatric Surgery YaAn People's Hospital Ya'an Sichuan China

**Keywords:** gastric cancer, myosteatosis, peripheral lymphoid subpopulation, prognosis, surgery

## Abstract

**Background:**

This study aimed to investigate the predictive capacity of lymphocyte subpopulations, sarcopenia and myosteatosis for clinical outcomes in patients who underwent gastric cancer surgery. Additionally, the prognostic significance of CD3+/CD4+ cells in conjunction with myosteatosis was explored.

**Methods:**

A cohort of 190 patients with gastric cancer who underwent surgery and received computed tomography scans between July 2016 and December 2017 at our institution was examined. Complete clinical information and peripheral lymphocyte subpopulations were available for all patients. A comprehensive array of statistical methodologies was employed to scrutinize variances in both clinical and pathological characteristics among patients, with the aim of identifying autonomous prognostic determinants requisite for the development of a nomogram. Subsequent assessment of the predictive efficacy of the nomogram was conducted via calibration curve analysis.

**Results:**

The study comprised a cohort of 190 participants, encompassing 126 males (66.32%) and 64 females (33.68%), with a mean age of 58.47 (±11.37) years. Patients were stratified into three groups based on CD3+/CD4+ cells and myosteatosis, with 24 in Group 1, 87 in Group 2 and 79 in Group 3. Notably, patients in the third group exhibited significantly shorter progression‐free survival (PFS) (hazard ratio [HR] = 0.208, *P* < 0.001) and overall survival (OS) (HR = 0.193, *P* < 0.001). The subset of peripheral blood lymphocytes exhibited elevated levels of CD3+/CD4+ cells (HR = 2.485, *P* < 0.001) and heightened CD4+/CD8+ ratios (HR = 1.705, *P* = 0.038), whereas diminished CD19+ cell counts (HR = 0.210, *P* = 0.032) correlated with improved OS in patients. The individuals presenting with sarcopenia (HR = 4.089, *P* = 0.023) and myosteatosis (HR = 2.857, *P* < 0.001) displayed reduced OS. The multivariate Cox regression analysis showed that pathological tumour–node–metastasis stage, CD19+ cells, sarcopenia and CD3+/CD4+ cell–myosteatosis were identified as independent prognostic factors for PFS and OS in patients. The constructed nomograms for PFS and OS yielded C‐index values of 0.839 (95% confidence interval [CI]: 0.798–0.880) and 0.836 (95% CI: 0.792–0.879), respectively. The calibration analysis demonstrated that the nomograms accurately predicted the 3‐ and 5‐year survival rates of PFS and OS in patients.

**Conclusions:**

Lymphocyte subsets, including CD3+/CD4+ cells, CD4+/CD8+ ratio and CD19+ cells, are indicative of clinical prognosis in gastric cancer surgery patients. Body composition parameters, such as sarcopenia and myosteatosis, are also associated with the patient's prognosis. The combination of CD3+/CD4+ cells with myosteatosis demonstrates enhanced prognostic value, enabling the identification of patients at high risk of post‐operative metastasis and recurrence.

## Introduction

Gastric cancer stands as a formidable public health menace, ranking fifth among the most prevalent malignant tumours and occupying the fourth position in terms of cancer‐related fatalities globally.^1^ Despite the array of treatment modalities available, surgical intervention remains the primary therapeutic avenue for individuals afflicted with gastric cancer. Nevertheless, even with radical resection, patients face a considerable risk of recurrence and metastasis.^2^, ^3^ Consequently, an imperative exists to investigate novel prognostic indicators that can facilitate the precise selection of treatment strategies for patients.

The immune system constitutes a pivotal component in impeding tumorigenesis and resisting tumour progression.^4^ T and B lymphocytes, in particular, assume crucial roles in immune surveillance, contributing substantially to the eradication of tumour cells.^5^ ^,^ ^S1^ Upon detecting aberrant cells, the immune system initiates an intricate cascade of cellular and molecular signals, activating immune cells to mount a response and ultimately eliminate these abnormal cells.^6^, ^7^ Hence, individuals with compromised immune function are predisposed to heightened risks of tumour recurrence and metastasis.^8^ Previous investigations have demonstrated that circulating lymphoid subpopulations portend an unfavourable prognosis in gastric cancer^9^ ^,^ ^S2^ and various other solid tumours.^10^ ^,^ ^S3^

Numerous studies have also emphasized the utility of body composition as a prognostic predictor in individuals with tumours.^11^ While body mass index (BMI) stands as the most commonly employed metric, its consistency with clinical outcomes in tumour patients has been shown to be variable.^12^ Significantly, BMI fails to consider the distribution of adipose tissue or the mass and quantity of skeletal muscle. Myosteatosis, characterized by abnormal adipose tissue distribution within and between muscle cells, results in excessive fat deposition within the muscle, which is a pathological state linked to diminished muscle mass, limb functionality and physical performance. Assessment of myosteatosis is conventionally conducted via skeletal muscle radiation attenuation (SMRA) on radiological computed tomography (CT) images.^13^ ^,^ ^S4^ Severe myosteatosis has been associated with an unfavourable prognosis in gastric cancer^14^ and other malignancies.^15^

The reciprocal interplay between the immune system and tissue wasting collectively contributes to tumour progression. Cancer‐associated tissue wasting and weight loss primarily arise from factors such as anorexia, inflammation and the release of tumour‐secreted molecules, which elevate energy expenditure and precipitate a state of metabolic disruption within the organism.^16^ ^,^ ^S5^ ^,^ ^S6^ Prolonged excessive energy consumption can induce malnutrition, consequently compromising immune function, diminishing resistance to tumours and facilitating their proliferation.^17^ ^,^ ^S7^

This study endeavours to elucidate the correlation between lymphoid subpopulations, severe myosteatosis and the prognosis of gastric cancer patients who underwent surgery. Moreover, the study aims to amalgamate CD3+/CD4+ cells with severe myosteatosis to formulate a novel and more comprehensive prognostic index with the intent of accurately appraising the recurrence and progression risks in gastric cancer patients.

## Materials and methods

### Patient cohort

This retrospective study was conducted with the approval of the Ethics Committee of Harbin Medical University Cancer Hospital, rendering the need for informed consent unnecessary. The investigation involved a cohort of 190 consecutive patients diagnosed with gastric cancer who underwent surgical procedures at Harbin Medical University Cancer Hospital between July 2016 and December 2017. These patients also underwent lymphatic subset testing. The analysis of data related to the 190 patients and their clinical profiles adhered to the principles outlined in the Helsinki Declaration and its subsequent amendments. The inclusion criteria stipulated that all patients must have undergone surgical intervention, undergone lymphatic subset testing and received abdominal CT scans at Harbin Medical University Cancer Hospital. All CT scans were performed 30 days before surgery. A standard radical gastrectomy consists of a gastrectomy with D2 lymphadenectomy and resection of N1 and N2 lymph nodes, as defined by the Japanese Classification of Gastric Carcinoma.^18^ Conversely, exclusion criteria comprised patients with chronic ailments, those exhibiting an acute inflammatory response, individuals concurrently diagnosed with other primary malignancies in addition to gastric cancer and those with incomplete clinical data. The flow chart for clinical case selection is shown in *Figure* *S1*. Comprehensive clinical and pathological information was gathered and stored in the electronic medical records system.

### Data acquisition

Patients were subjected to follow‐up assessments through telephone consultations or outpatient visits. These follow‐ups occurred every 3–6 months during the initial 2 years post‐surgery, followed by intervals of every 6–12 months for the third to fifth years and subsequently on an annual basis. Progression‐free survival (PFS) was calculated from the date of surgery initiation to the occurrence of disease progression, mortality, cessation of follow‐up or the final follow‐up occasion. Disease progression was identified through chest and abdominal X‐rays or CT. Overall survival (OS) was defined as the interval from the date of surgery initiation to mortality, cessation of follow‐up or the last follow‐up occasion. The electronic medical records system served as the repository for compiling patients' clinical and pathological data.

### Evaluation of sarcopenia and myosteatosis

A single radiologist, who remained unaware of clinical information, conducted the interpretation of CT scans utilizing imaging software to assess skeletal muscle density (SMD) and additional body composition metrics. Following this, two separate researchers, operating independently, conducted an audit of the initial estimations by reassessing a randomly selected 30% subset of CT images. The outcomes of this auditing process consistently fell within a margin of ±5.0%. Subsequently, the CT data for each patient were imported into 3D Slicer (Version 4.10.2, www.slicer.org) for a comprehensive assessment of the cross‐sectional area of skeletal muscles at the third lumbar vertebra (L3) and the mean SMD, measured in Hounsfield units (HU) across the entire muscle region. The HU thresholds for skeletal muscle were set within the range of −29 to 150. The skeletal muscle index (SMI) for L3 was computed as the ratio of skeletal muscle area (in square centimetres) to the square of the patient's height (in square metres). Concurrently, peripheral lymphatic subset levels were systematically quantified and analysed through a dedicated protein analyser (BD FACSCanto).

Optimal cut‐off points for lymphatic subsets were determined via receiver operating characteristic (ROC) analysis, with OS serving as the predictive endpoint for patient mortality. The area under the ROC curve (AUC) was employed to assess the predictive capacity of lymphatic subsets. Sex‐specific SMI cut‐off values of 42.2 cm^2^/m^2^ for men and 33.9 cm^2^/m^2^ for women were used to define sarcopenia.^19^ Myosteatosis was defined using the mean SMD, with a cut‐off value of <41 HU for patients with a BMI of <25 kg/m^2^ and <33 HU for those with a BMI of 25 kg/m^2^ or higher.^20^ Patients with CD3+/CD4+ cell ≥ 42.05% and myosteatosis comprised Group 1, while patients with CD3+/CD4+ cell < 42.05% and without myosteatosis were categorized under Group 3. The remaining cases were placed in Group 2.

### Statistical analysis

Continuous variables were presented as means with standard deviations or as medians with interquartile ranges. Categorical variables were expressed as percentages. One‐way analysis of variance (ANOVA), Kruskal–Wallis rank sum test and chi‐square test or Fisher's exact test were utilized for comparisons between continuous and categorical variables, respectively. Kaplan–Meier survival curves and log‐rank tests were employed to compute and compare survival rates and times. Utilizing the Cox proportional hazards model, univariate analysis was conducted, taking into account factors demonstrating significant prognostic relevance with a *P* value below 0.05. All identified prognostic factors underwent systematic optimization based on Akaike information criterion (AIC) values to prevent overfitting. Variables optimized through stepwise regression were subsequently integrated into the multifactor Cox proportional hazards model analysis to ascertain independent prognostic factors for both PFS and OS. Hazard ratios (HRs) and 95% confidence intervals (CIs) were used to assess relative risks. Nomograms were constructed to predict 1‐, 3‐ and 5‐year survival probabilities for PFS and OS. Calibration curve analysis was employed to assess the prognostic predictive ability of the nomogram. All statistical analyses were performed using R 4.1.3 (Vienna, Austria), GraphPad Prism 8.0 (La Jolla, CA, USA) and IBM SPSS Statistics 25 (Chicago, IL, USA), with two‐sided *P* values < 0.05 considered statistically significant.

## Results

### Patient characteristics

A total of 190 patients diagnosed with gastric cancer were enrolled in this study, comprising 126 males and 64 females, with a median age of 60 years. All patients underwent surgical intervention, and 185 individuals underwent radical resection. Chi‐square and Fisher's exact tests revealed significant associations between CD3+/CD4+ cell–myosteatosis and sarcopenia (*P* < 0.001). Furthermore, one‐way ANOVA and Kruskal–Wallis rank sum tests unveiled noteworthy associations between CD3+/CD4+ cell–myosteatosis and several variables, including age, alanine aminotransferase (ALT), total bilirubin (TBIL), prealbumin (PALB), red blood cell count (RBC), platelet count (P), carbohydrate antigen 724 (CA724), CD3+ cells (T cells), CD3+/CD8+ cells (CTL cells), CD4+/CD8+ ratio and CD3−/CD16+CD56+ cells (natural killer [NK] cells), all exhibiting *P* values < 0.05. A comprehensive tabulation of clinical characteristics and blood indicators for all 190 cases, categorized by CD3+/CD4+ cell–myosteatosis, is provided in *Table* *1*.

**Table 1:** Clinical, pathological and laboratory information of all patients

Item	Level	Group 1	Group 2	Group 3	*P*
		24	87	79	
Age	Median (IQR)	64.00 (59.25–70.50)	62.00 (51.00–68.00)	57.00 (47.00–62.00)	<0.001
BMI	Mean ± SD	22.32 ± 2.22	22.11 ± 3.16	22.62 ± 3.68	0.526
Sex	Male	15 (62.5)	50 (57.5)	61 (77.2)	0.025
	Female	9 (37.5)	37 (42.5)	18 (22.8)	
Stomach ache	No	9 (37.5)	21 (24.1)	18 (22.8)	0.330
	Yes	15 (62.5)	66 (75.9)	61 (77.2)	
Melaena	No	20 (83.3)	65 (74.7)	63 (79.7)	0.582
	Yes	4 (16.7)	22 (25.3)	16 (20.3)	
Radical resection	No	2 (8.3)	3 (3.4)	0 (0.0)	0.067
	Yes	22 (91.7)	84 (96.6)	79 (100.0)	
Tumour size	<50 mm	12 (50.0)	40 (46.0)	46 (58.2)	0.284
	≥50 mm	12 (50.0)	47 (54.0)	33 (41.8)	
pTNM stage	Tis/0 + I + II	14 (58.3)	51 (58.6)	59 (74.7)	0.071
	III + IV	10 (41.7)	36 (41.4)	20 (25.3)	
ALT (U/L)	Median (IQR)	15.00 (10.20–25.50)	16.00 (13.00–21.00)	20.00 (14.00–26.00)	0.033
LDH (U/L)	Median (IQR)	159.50 (138.25–181.25)	164.00 (147.00–185.00)	153.00 (142.00–176.00)	0.132
TBIL (μmol/L)	Median (IQR)	13.72 (8.70–16.62)	9.92 (7.88–13.04)	12.70 (9.39–16.59)	0.001
TP (g/L)	Median (IQR)	67.80 (61.85–70.75)	68.00 (64.00–71.80)	68.00 (64.00–73.00)	0.778
ALB (g/L)	Median (IQR)	40.00 (37.13–42.40)	40.10 (38.00–42.00)	41.00 (38.00–44.00)	0.320
PALB (mg/L)	Mean ± SD	234.54 ± 70.65	257.60 ± 61.37	283.86 ± 81.08	0.005
Urea (mmol/L)	Median (IQR)	4.90 (4.23–6.30)	5.50 (4.40–6.70)	5.80 (5.10–6.70)	0.075
WBC (10^9^/L)	Mean ± SD	7.13 ± 3.23	6.19 ± 1.59	7.06 ± 2.46	0.093
NEU (10^9^/L)	Mean ± SD	4.54 ± 3.35	3.65 ± 1.14	4.32 ± 2.41	0.505
L (10^9^/L)	Median (IQR)	1.93 (1.42–2.33)	1.81 (1.38–2.35)	2.01 (1.57–2.57)	0.317
Mono (10^9^/L)	Median (IQR)	0.49 (0.36–0.65)	0.43 (0.31–0.57)	0.46 (0.36–0.57)	0.424
RBC (10^12^/L)	Median (IQR)	4.37 (0.04–476)	4.24 (3.92–.456)	4.63 (4.20–5.04)	<0.001
P (10^9^/L)	Median (IQR)	224.00 (167.75–251.00)	260.00 (246.00–313.00)	249.00 (203.00–306.00)	0.02
CEA (ng/mL)	Median (IQR)	1.67 (1.17–4.08)	2.00 (1.32–4.39)	1.94 (0.94–3.58)	0.374
CA724 (U/mL)	Median (IQR)	10.85 (7.57–37.77)	9.68 (5.51–19.44)	7.91 (3.44–14.76)	0.02
CA199 (U/mL)	Median (IQR)	2.13 (1.35–9.73)	3.03 (1.17–6.89)	1.69 (1.03–3.55)	0.073
CA125II (U/mL)	Median (IQR)	10.75 (6.32–15.22)	10.87 (7.43–16.68)	9.27 (6.44–12.41)	0.102
CD3+ cell (%)	Median (IQR)	73.75 (68.35–76.78)	71.50 (65.70–77.70)	67.90 (59.90–73.30)	0.004
CD/CD8+ cell (%)	Mean ± SD	18.85 ± 6.51	21.97 ± 6.81	25.51 ± 7.45	<0.001
CD4+/CD8+ ratio	Median (IQR)	2.87 (2.24–3.51)	2.02 (1.45–2.77)	1.44 (1.23–1.85)	<0.001
CD3+/CD4+CD8+ cell (%)	Median (IQR)	0.30 (0.10–0.475)	0.20 (0.10–0.50)	0.30 (0.20–0.59)	0.135
CD19+ cell (%)	Mean ± SD	10.89 ± 5.18	10.87 ± 4.82	10.63 ± 4.56	0.942
CD3−/CD16+CD56+ cell (%)	Median (IQR)	13.20 (8.93–17.80)	13.80 (8.80–20.80)	17.60 (11.30–25.30)	0.011
CD3+/CD16+CD56+ cell (%)	Median (IQR)	1.30 (0.70–2.65)	1.90 (0.90–4.30)	1.80 (1.10–350)	0.297
SAT (cm^2^)	Median (IQR)	87.68 (47.223–109.74)	78.73 (56.95–124.55)	75.33 (42.72–115.32)	0.668
VAT (cm^2^)	Median (IQR)	63.54 (32.04–85.29)	63.13 (29.96–87.44)	69.65 (23.71–107.09)	0.953
Sarcopenia	No	6 (25.0)	26 (29.9)	45 (57.0)	<0.001
	Yes	18 (75.0)	61 (70.1)	34 (43.0)	

*Note*: Group 1: CD3+/CD4+ cell ≥ 42.05% and myosteatosis; Group 2: CD3+/CD4+ cell ≥ 42.05% and without myosteatosis, or CD3+/CD4+ cell < 42.05% and without myosteatosis; and Group 3: CD3+/CD4+ cell < 42.05% and without myosteatosis. Abbreviations: ALB, albumin; ALT, alanine aminotransferase; BMI, body mass index; CA125II, carbohydrate antigen 125II; CA199, carbohydrate antigen 199; CA724, carbohydrate antigen 724; CEA, carcinoembryonic antigen; IQR, interquartile range; L, lymphocyte; LDH, lactate dehydrogenase; Mono, monocyte; NEU, neutrophil; P, platelet; PALB, prealbumin; pTNM, pathological tumour–node–metastasis; RBC, red blood cell count; SAT, subcutaneous adipose tissue; SD, standard deviation; TBIL, total bilirubin; TP, total protein; VAT, visceral adipose tissue; WBC, white blood cell count.

### Univariate and multivariate Cox hazard analysis for progression‐free survival and overall survival

Univariate analysis identified several prognostic factors for patients' PFS in this study, including age (HR = 2.323, 95% CI: 1.324–4.073, *P* = 0.003), CA724 (HR = 2.224, 95% CI: 1.317–3.810, *P* = 0.003), CD19+ cells (HR = 0.157, 95% CI: 0.038–0.643, *P* = 0.010), tumour size (HR = 3.078, 95% CI: 1.784–5.308, *P* < 0.001), pathological tumour–node–metastasis (pTNM) stage (HR = 2.323, 95% CI: 1.324–4.073, *P* < 0.001), subcutaneous adipose tissue (SAT) (HR = 0.399, 95% CI: 0.181–0.878, *P* = 0.022), visceral adipose tissue (VAT) (HR = 0.292, 95% CI: 0.125–0.668, *P* = 0.004), sarcopenia (HR = 0.108, 95% CI: 0.034–0.345, *P* < 0.001) and CD3+/CD4+ cell–myosteatosis (*P* < 0.001). Similarly, for OS, prognostic factors identified through univariate analysis included age (HR = 2.284, 95% CI: 1.317–3.960, *P* = 0.003), CA724 (HR = 2.274, 95% CI: 1.340–3.859, *P* = 0.002), CD4+/CD8+ ratio (HR = 1.705, 95% CI: 1.030–2.821, *P* = 0.038), CD19+ cells (HR = 0.152, 95% CI: 0.037–0.622, *P* = 0.009), tumour size (HR = 3.099, 95% CI: 1.800–5.333, *P* < 0.001), pTNM stage (HR = 6.618, 95% CI: 3.381–11.431, *P* < 0.001), SAT (HR = 0.392, 95% CI: 0.178–0.861, *P* = 0.020), VAT (HR = 0.287, 95% CI: 0.124–0.668, *P* = 0.004), sarcopenia (HR = 0.110, 95% CI: 0.034–0.351, *P* < 0.001) and CD3+/CD4+ cell–myosteatosis (*P* < 0.001). These variables underwent optimization through the assessment of AIC values to mitigate the risk of overfitting. The optimal models for PFS and OS encompassed CA724, pTNM stage, CD19+ cells, VAT, sarcopenia and CD3+/CD4+ cell–myosteatosis. Multivariate analysis further elucidated independent prognostic factors for PFS, highlighting that pTNM stage (HR = 4.956, 95% CI: 2.797–8.781, *P* < 0.001), CD19+ cells (HR = 0.230, 95% CI: 0.055–0.961, *P* = 0.044), sarcopenia (HR = 4.343, 95% CI: 1.285–14.678, *P* = 0.018) and CD3+/CD4+ cell–myosteatosis (*P* < 0.001) were significant contributors (*Table* *2*). In the case of OS, multivariate analysis identified pTNM stage (HR = 4.830, 95% CI: 2.741–8.511, *P* < 0.001), CD19+ cells (HR = 0.210, 95% CI: 0.051–0.876, *P* = 0.032), sarcopenia (HR = 4.089, 95% CI: 1.214–13.832, *P* = 0.023) and CD3+/CD4+ cell–myosteatosis (*P* < 0.001) as independent prognostic factors (*Table* *3*).

**Table 2:** Univariate and multivariate analysis for progression‐free survival

Parameters	Univariate analysis	*P* value	Multivariate analysis	*P* value
	Hazard ratio (95% CI)		Hazard ratio (95% CI)	
Sex (male vs. female)	1.119 (0.644–1.944)	0.690		
Age (<60 vs. ≥60)	2.323 (1.324–4.073)	0.003		
BMI (<22.07 vs. ≥22.07 kg/m^2^)	0.699 (0.419–1.167)	0.171		
Stomach ache (no vs. yes)	1.686 (0.877–3.244)	0.118		
Melaena (no vs. yes)	1.581 (0.910–2.747)	0.104		
Tumour size (<50 vs. ≥50 mm + unknown)	3.078 (1.784–5.308)	<0.001		
pTNM (0/Tis + I + II vs. III + IV)	6.983 (4.027–12.108)	<0.001	4.956 (2.797–8.781)	<0.001
ALT (<17 vs. ≥17 U/L)	0.775 (0.467–1.288)	0.325		
LDH (<160.5 vs. ≥160.5 U/L)	1.480 (0.888–2.467)	0.133		
TBIL (<11.02 vs. ≥11.02 μmol/L)	0.695 (0.417–1.158)	0.163		
TP (<68 vs. ≥68 g/L)	1.069 (0.644–1.774)	0.796		
ALB (<41 vs. ≥41 g/L)	0.834 (0.503–1.385)	0.484		
PALB (<264.5 vs. ≥264.5 mg/L)	0.642 (0.384–1.073)	0.091		
Urea (<5.65 vs. ≥5.65 mmol/L)	1.203 (0.724–2.001)	0.476		
WBC (<6.39 vs. ≥6.39 10^9^/L)	0.802 (0.483–1.335)	0.397		
NEU (<3.60 vs. ≥3.60 10^9^/L)	1.002 (0.604–1.662)	0.995		
Mono (<0.44 vs. ≥0.44 10^9^/L)	1.087 (0.652–1.812)	0.749		
RBC (<4.38 vs. ≥4.38 10^12^/L)	0.674 (0.404–1.127)	0.133		
P (<252 vs. ≥252 10^9^/L)	0.999 (0.602–1.658)	0.998		
CEA (<1.98 vs. ≥1.98 ng/mL)	1.417 (0.850–2.362)	0.181		
CA199 (<9.43 vs. ≥9.43 U/mL)	1.663 (0.992–2.788)	0.054		
CA724 (<2.10 vs. ≥2.10 U/mL)	2.224 (1.317–3.810)	0.003	1.518 (0.882–2.612)	0.132
CA125II (<9.80 vs. ≥9.80 U/mL)	1.162 (0.964–2.693)	0.068		
CD3+ cell (<73.50% vs. ≥73.50%)	1.533 (0.920–2.555)	0.101		
CD3+/CD4+ cell (<42.05% vs. ≥42.05%)	2.440 (1.470–4.051)	0.001		
CD3+/CD8+ cell (<26.55% vs. ≥26.55%)	0.608 (0.339–1.089)	0.094		
CD4+/CD8+ ratio (<1.95 vs. ≥1.95)	1.646 (0.995–2.723)	0.052		
CD3+/CD4+CD8+ cell (<0.45% vs. ≥0.45%)	0.639 (0.351–1.163)	0.142		
CD19+ cell (<14.85% vs. ≥14.85%)	0.157 (0.038–0.643)	0.010	0.230 (0.055–0.961)	0.044
CD3−/CD16+CD56+ cell (<14.65% vs. ≥14.65%)	0.728 (0.438–1.212)	0.222		
CD3+/CD16+CD56+ cell (<6.65% vs. ≥6.65%)	2.000 (0.949–4.214)	0.068		
SAT (<119.73 vs. ≥119.73 cm^2^)	0.399 (0.181–0.878)	0.022		
VAT (<100.14 vs. ≥100.14 cm^2^)	0.292 (0.125–0.668)	0.004	0.475 (0.201–1.124)	0.090
Sarcopenia (no vs. yes)	9.256 (2.898–29.561)	<0.001	4.343 (1.285–14.678)	0.018
Myosteatosis (no vs. yes)	2.907 (1.756–4.811)	<0.001		
CD3+/CD4+ cell–myosteatosis				
Group 1	Ref		Ref	
Group 2	0.454 (0.246–0.836)	0.011	0.399 (0.215–0.739)	0.003
Group 3	0.129 (0.059–0.283)	<0.001	0.208 (0.092–0.471)	<0.001

*Note*: Group 1: CD3+/CD4+ cell ≥ 42.05% and myosteatosis; Group 2: CD3+/CD4+ cell ≥ 42.05% and without myosteatosis, or CD3+/CD4+ cell < 42.05% and without myosteatosis; and Group 3: CD3+/CD4+ cell < 42.05% and without myosteatosis. Abbreviations: ALB, albumin; ALT, alanine aminotransferase; BMI, body mass index; CA125II, carbohydrate antigen 125II; CA199, carbohydrate antigen 199; CA724, carbohydrate antigen 724; CEA, carcinoembryonic antigen; CI, confidence interval; LDH, lactate dehydrogenase; Mono, monocyte; NEU, neutrophil; P, platelet; PALB, prealbumin; pTNM, pathological tumour–node–metastasis; RBC, red blood cell count; Ref, reference; SAT, subcutaneous adipose tissue; TBIL, total bilirubin; TP, total protein; VAT, visceral adipose tissue; WBC, white blood cell count.

**Table 3:** Univariate and multivariate analysis for overall survival

Parameters	Univariate analysis	*P* value	Multivariate analysis	*P* value
	Hazard ratio (95% CI)		Hazard ratio (95% CI)	
Sex (male vs. female)	0.905 (0.527–1.557)	0.719		
Age (<60 vs. ≥60)	2.284 (1.317–3.960)	0.003		
BMI (<22.07 vs. ≥22.07 kg/m^2^)	0.692 (0.416–1.150)	0.155		
Stomach ache (no vs. yes)	1.753 (0.912–3.369)	0.092		
Melaena (no vs. yes)	1.627 (0.939–2.822)	0.083		
Tumour size (<50 vs. ≥50 mm + unknown)	3.099 (1.800–5.333)	<0.001		
pTNM (0/Tis + I + II vs. III + IV)	6.618 (3.831–11.431)	<0.001	4.830 (2.741–8.511)	<0.001
ALT (<17 vs. ≥17 U/L)	0.766 (0.463–1.267)	0.299		
LDH (<160.5 vs. ≥160.5 U/L)	1.423 (0.859–2.360)	0.171		
TBIL (<11.02 vs. ≥11.02 μmol/L)	0.663 (0.399–1.103)	0.113		
TP (<68 vs. ≥68 g/L)	1.015 (0.614–1.676)	0.955		
ALB (<41 vs. ≥41 g/L)	0.786 (0.475–1.300)	0.348		
PALB (<264.5 vs. ≥264.5 mg/L)	0.629 (0.377–1.048)	0.075		
Urea (<5.65 vs. ≥5.65 mmol/L)	1.195 (0.722–1.977)	0.489		
WBC (<6.39 vs. ≥6.39 10^9^/L)	0.798 (0.482–1.323)	0.383		
NEU (<3.60 vs. ≥3.60 10^9^/L)	1.016 (0.615–1.678)	0.951		
Mono (<0.44 vs. ≥0.44 10^9^/L)	1.136 (0.683–1.887)	0.623		
RBC (<4.38 vs. ≥4.38 10^12^/L)	0.660 (0.396–1.099)	0.110		
P (<252 vs. ≥252 10^9^/L)	1.071 (0.648–1.769)	0.790		
CEA (<1.98 vs. ≥1.98 ng/mL)	1.547 (0.931–2.572)	0.092		
CA199 (<9.43 vs. ≥9.43 U/mL)	1.642 (0.985–2.737)	0.057		
CA724 (<2.10 vs. ≥2.10 U/mL)	2.274 (1.340–3.859)	0.002	1.487 (0.863–2.563)	0.153
CA125II (<9.80 vs. ≥9.80 U/mL)	1.613 (0.938–2.687)	0.067		
CD3+ cell (<73.50% vs. ≥73.50%)	1.541 (0.929–2.555)	0.094		
CD3+/CD4+ cell (<42.05% vs. ≥42.05%)	2.485 (1.496–4.129)	<0.001		
CD3+/CD8+ cell (<26.55% vs. ≥26.55%)	0.590 (0.329–1.057)	0.076		
CD4+/CD8+ ratio (<1.95 vs. ≥1.95)	1.705 (1.030–2.821)	0.038		
CD3+/CD4+CD8+ cell (<0.45% vs. ≥0.45%)	0.643 (0.354–1.168)	0.147		
CD19+ cell (<14.85% vs. ≥14.85%)	0.152 (0.037–0.622)	0.009	0.210 (0.051–0.876)	0.032
CD3−/CD16+CD56+ cell (<14.65% vs. ≥14.65%)	0.737 (0.445–1.222)	0.237		
CD3+/CD16+CD56+ cell (<6.65% vs. ≥6.65%)	1.986 (0.943–4.182)	0.071		
SAT (<119.73 vs. ≥119.73 cm^2^)	0.392 (0.178–0.861)	0.020		
VAT (<100.14 vs. ≥100.14 cm^2^)	0.287 (0.124–0.668)	0.004	0.422 (0.178–1.000)	0.050
Sarcopenia (no vs. yes)	9.088 (2.846–29.021)	<0.001	4.089 (1.214–13.832)	0.023
Myosteatosis (no vs. yes)	2.857 (1.727–4.726)	<0.001		
CD3+/CD4+ cell–myosteatosis				
Group 1	Ref		Ref	
Group 2	0.463 (0.252–0.851)	0.013	0.400 (0.216–0.741)	0.004
Group 3	0.128 (0.059–0.282)	<0.001	0.193 (0.085–0.439)	<0.001

*Note*: Group 1: CD3+/CD4+ cell ≥ 42.05% and myosteatosis; Group 2: CD3+/CD4+ cell ≥ 42.05% and without myosteatosis, or CD3+/CD4+ cell < 42.05% and without myosteatosis; and Group 3: CD3+/CD4+ cell < 42.05% and without myosteatosis. Abbreviations: ALB, albumin; ALT, alanine aminotransferase; BMI, body mass index; CA125II, carbohydrate antigen 125II; CA199, carbohydrate antigen 199; CA724, carbohydrate antigen 724; CEA, carcinoembryonic antigen; CI, confidence interval; LDH, lactate dehydrogenase; Mono, monocyte; NEU, neutrophil; P, platelet; PALB, prealbumin; pTNM, pathological tumour–node–metastasis; RBC, red blood cell count; Ref, reference; SAT, subcutaneous adipose tissue; TBIL, total bilirubin; TP, total protein; VAT, visceral adipose tissue; WBC, white blood cell count.

### Survival analysis for lymphocyte subsets

Utilizing Cox regression analysis, we identified significant associations between certain lymphatic subgroup indicators and the prognostic outcomes of patients with gastric cancer. The determination of optimal cut‐off values for CD3+/CD4+ cells, CD4+/CD8+ ratio and CD19+ cells through ROC curve analysis yielded values of 42.05%, 1.95 and 14.85%, respectively. Subsequently, patients were stratified based on these cut‐off values, revealing distinct survival rates and HRs for PFS and OS.

**Figure 1:**
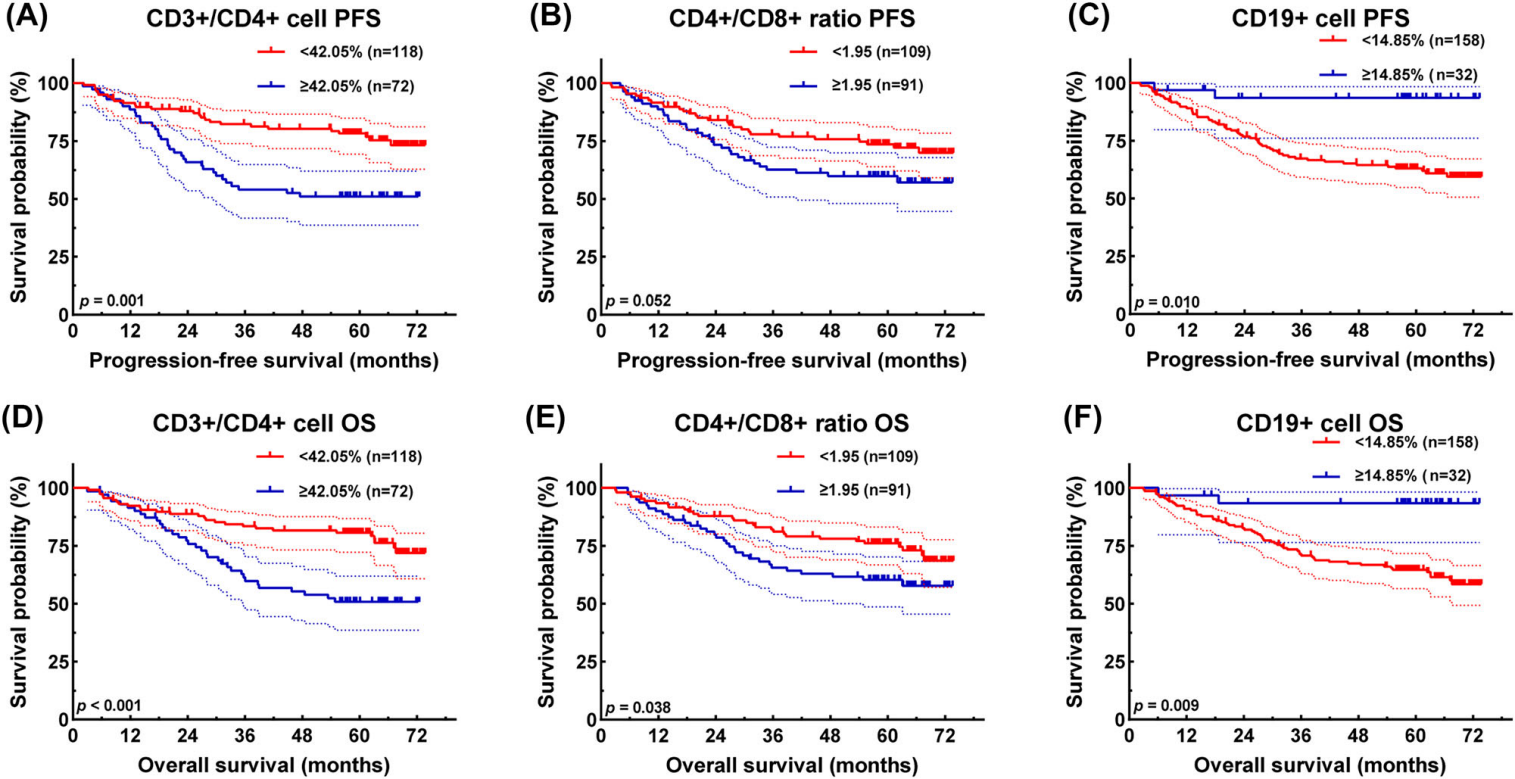
Survival curve for the lymphocyte subset. CD3+/CD4+ cell‐related survival curve for (A) progression‐free survival (PFS) and (D) overall survival (OS); CD4+/CD8+ ratio‐related survival curve for (B) PFS and (E) OS; and CD19+ cell‐related survival curve for (C) PFS and (F) OS.

For CD3+/CD4+ cells, 118 patients exhibited levels below 42.05%, with 1‐, 3‐ and 5‐year PFS rates of 91.5% (95% CI: 86.6–96.4%), 82.3% (95% CI: 75.6–89.7%) and 78.3% (95% CI: 70.9–86.4%), and corresponding OS rates of 92.3% (95% CI: 87.7–97.3%), 83.5% (95% CI: 77.0–90.6%) and 80.8% (95% CI: 73.8–88.3%). Conversely, 72 patients with CD3+/CD4+ levels ≥ 42.05% exhibited 1‐, 3‐ and 5‐year PFS rates of 88.7% (95% CI: 81.7–96.4%), 54.0% (95% CI: 43.5–67.2%) and 51.0% (95% CI: 40.4–64.3%), and corresponding OS rates of 91.6% (95% CI: 85.3–98.3%), 61.3% (95% CI: 50.9–73.9%) and 50.9% (95% CI: 40.3–64.2%). Elevated CD3+/CD4+ cell levels were associated with poorer PFS (HR = 2.440, 95% CI: 1.470–4.051, *P* = 0.001) and OS (HR = 2.485, 95% CI: 1.496–4.129, *P* < 0.001) (*Figure* *1* *A,D*).

For the CD4+/CD8+ ratio, 109 cases fell into the <1.95 group, with 1‐, 3‐ and 5‐year PFS rates of 91.7% (95% CI: 86.7–97.15%), 78.0% (95% CI: 70.4–86.4%) and 73.7% (95% CI: 65.6–82.8%), and corresponding OS rates of 93.6% (95% CI: 89.1–98.3%), 82.1% (95% CI: 75.2–89.8%) and 76.2% (95% CI: 68.4–84.8%). Conversely, 81 cases with CD4+/CD8+ ratio ≥ 1.95 exhibited 1‐, 3‐ and 5‐year PFS rates of 88.8% (95% CI: 82.1–96.0%), 62.6% (95% CI: 52.7–74.4%) and 59.9% (95% CI: 49.8–71.9%), and corresponding OS rates of 93.6% (95% CI: 89.1–98.3%), 82.1% (95% CI: 75.2–89.8%) and 76.2% (95% CI: 68.4–84.8%). Notably, a high CD4+/CD8+ ratio was associated with shorter PFS (HR = 1.646, 95% CI: 0.995–2.723, *P* = 0.052) and OS (HR = 1.705, 95% CI: 1.030–2.821, *P* = 0.038) (*Figure* *1* *B,E*).

For CD19+ cells, 158 patients had levels < 14.85%, with 1‐ and 3‐year PFS and OS rates of 89.2% (95% CI: 84.5–94.2%), 67.3% (95% CI: 60.3–75.2%) and 63.1% (95% CI: 55.8–71.3%), and 91.1% (95% CI: 86.7–95.6%), 71.5% (95% CI: 64.7–79.0%) and 64.7% (95% CI: 57.5–72.8%), respectively. In contrast, 72 patients with CD19+ levels ≥ 14.85% exhibited 1‐, 3‐ and 5‐year PFS and OS rates of 96.9% (95% CI: 91.0–100.0%), 93.4% (95% CI: 85.0–100.0%) and 93.4% (95% CI: 85.0–100.0%), and 96.9% (95% CI: 91.0–100.0%), 93.5% (95% CI: 85.2–100.0%) and 93.5% (95% CI: 85.2–100.0%), respectively. Patients with low CD19+ levels had inferior PFS (HR = 0.230, 95% CI: 0.055–0.0.961, *P* = 0.044) and OS (HR = 0.210, 95% CI: 0.051–0.876, *P* = 0.032) (*Figure* *1* *C,F*).

### Survival analysis for sarcopenia and myosteatosis

Utilizing Cox regression analysis, we discerned significant associations between body composition parameters and the prognostic outcomes of gastric cancer patients. Sex‐specific SMI cut‐off values of 42.2 cm^2^/m^2^ for men and 33.9 cm^2^/m^2^ for women were used to define sarcopenia.^19^ Stratifying patients based on these cut‐off values revealed distinct survival rates and HRs for PFS and OS.

**Figure 2:**
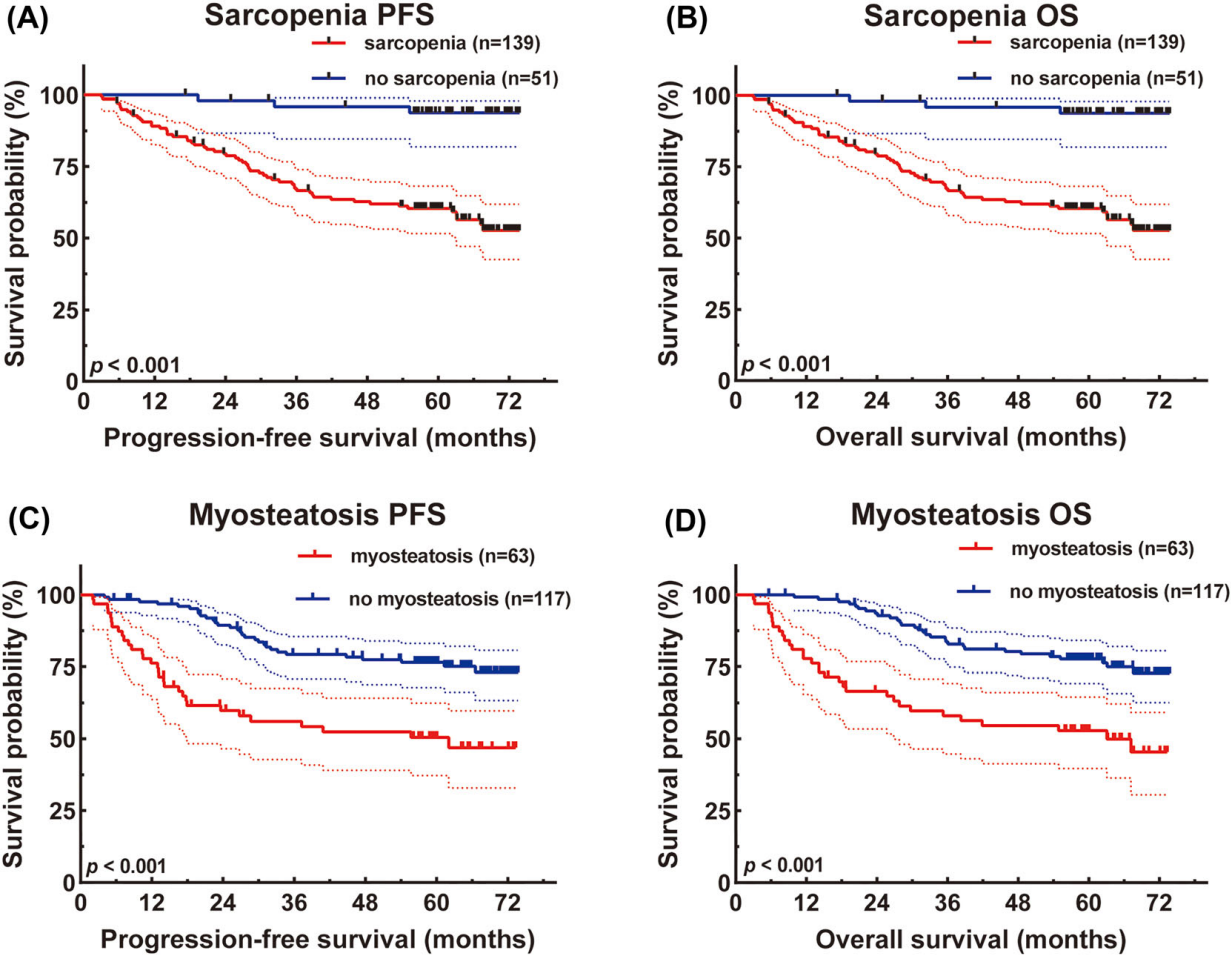
Sarcopenia‐related survival curve for (A) progression‐free survival (PFS) and (B) overall survival (OS), and myosteatosis‐related survival curve for (C) PFS and (D) OS.

A total of 139 patients suffered from sarcopenia, and 51 did not suffer from sarcopenia. The 1‐, 3‐ and 5‐year survival rates for PFS in the sarcopenia group were 87.0% (95% CI: 81.5–92.8%), 62.4% (95% CI: 54.6–71.2%) and 58.2% (95% CI: 50.3–67.3%), while for OS, these rates were 89.1% (95% CI: 84.1–94.5%), 67.4% (95% CI: 59.9–75.8%) and 60.4% (95% CI: 52.6–69.3%). The 1‐, 3‐ and 5‐year survival rates for PFS in patients without sarcopenia were 100.0% (95% CI: 100.0–100.0%), 95.9% (95% CI: 90.4–100.0%) and 93.6% (95% CI: 86.8–100.0%), while for OS, these rates were 100.0% (95% CI: 100.0–100.0%), 95.9% (95% CI: 90.5–100.0%) and 93.8% (95% CI: 87.2–100%). Patients with sarcopenia had poorer PFS (HR = 4.343, 95% CI: 1.285–14.678, *P* = 0.018) and OS (HR = 4.089, 95% CI: 1.214–13.832, *P* = 0.023) (*Figure* *2* *A,B*).

A total of 63 patients suffered from myosteatosis, and 127 did not suffer from myosteatosis. The 1‐, 3‐ and 5‐year survival rates for PFS in the myosteatosis group were 76.2% (95% CI: 66.4–87.5%), 56.1% (95% CI: 44.9–70.1%) and 50.5% (95% CI: 39.2–65.0%), while for OS, these rates were 77.8% (95% CI: 68.2–88.8%), 58.0% (95% CI: 46.9–71.8%) and 52.9% (95% CI: 41.7–67.0%). The 1‐, 3‐ and 5‐year survival rates for PFS in patients without myosteatosis were 97.6% (95% CI: 95.0–100.0%), 79.2% (95% CI: 72.3–86.8%) and 76.5% (95% CI: 69.2–84.5%), while for OS, these rates were 99.2% (95% CI: 97.7–100.0%), 83.6% (95% CI: 77.3–90.5%) and 77.7% (95% CI: 70.6–85.5%). Patients with myosteatosis had worse PFS (HR = 2.907, 95% CI: 1.756–4.811, *P* < 0.001) and OS (HR = 2.857, 95% CI: 1.727–4.726, *P* < 0.001) (*Figure* *2* *C,D*).

### CD3+/CD4+ cell–myosteatosis and prognosis

**Figure 3:**
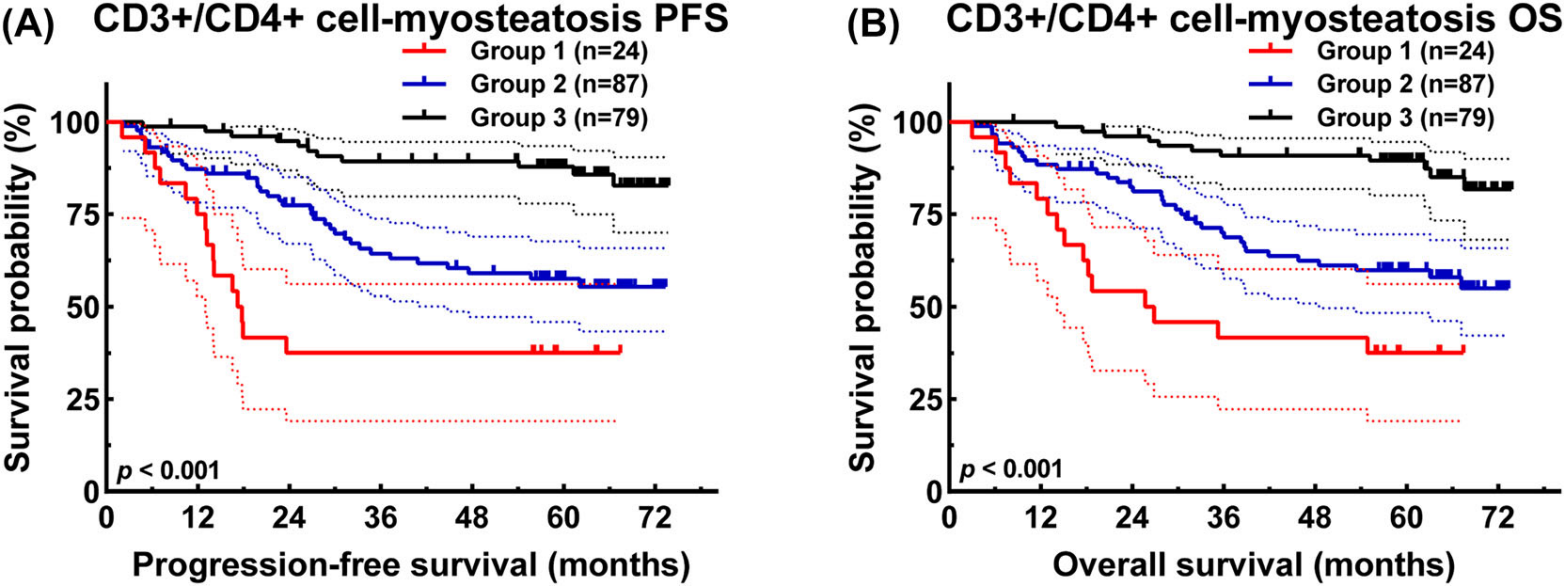
CD3+/CD4+ cell–myosteatosis‐related survival curve of (A) progression‐free survival (PFS) and (B) overall survival (OS) in all patients. Group 1: CD3+/CD4+ cell ≥ 42.05% and myosteatosis; Group 2: CD3+/CD4+ cell ≥ 42.05% and without myosteatosis, or CD3+/CD4+ cell < 42.05% and without myosteatosis; and Group 3: CD3+/CD4+ cell < 42.05% and without myosteatosis.

Patients were stratified into distinct groups, with Group 1 comprising 24 cases demonstrating 1‐, 3‐ and 5‐year survival rates for PFS of 75.0% (95% CI: 59.5–94.5%), 37.5% (95% CI: 22.4–62.9%) and 37.5% (95% CI: 22.4–62.9%), and corresponding rates for OS of 79.2% (95% CI: 64.5–97.2%), 41.7% (95% CI: 26.0–66.9%) and 37.5% (95% CI: 22.4–62.9%). Group 2, consisting of 87 cases, exhibited 1‐, 3‐ and 5‐year survival rates for PFS of 87.2% (95% CI: 80.4–94.6%), 64.3% (95% CI: 54.7–75.7%) and 57.5% (95% CI: 47.6–69.6%), and corresponding rates for OS of 88.4% (95% CI: 81.9–95.4%), 70.0% (95% CI: 60.8–80.6%) and 59.8% (95% CI: 50.0–71.5%). Lastly, Group 3, comprising 79 cases, demonstrated 1‐, 3‐ and 5‐year survival rates for PFS of 98.7% (95% CI: 96.3–100.0%), 89.4% (95% CI: 82.6–96.6%) and 87.8% (95% CI: 80.7–95.6%), and corresponding rates for OS of 100.0% (95% CI: 100.0–100.0), 90.9% (95% CI: 84.6–97.6%) and 89.5% (95% CI: 82.8–96.7%). Statistical analysis revealed that patients in Group 1 exhibited worse PFS (HR = 0.208, 95% CI: 0.092–0.471, *P* < 0.001) and OS (HR = 0.193, 95% CI: 0.085–0.439, *P* < 0.001) (*Figure* *3* *A,B*).

### Survival analysis for the pathological tumour–node–metastasis stage

To explore the association between the predictive capacity of the CD3+/CD4+ cell–myosteatosis in gastric cancer patients and the pTNM stage, we categorized 124 patients into an early pTNM stage (0/Tis + I + II) group and 66 patients into an advanced pTNM stage (III + IV) group. The 1‐, 3‐ and 5‐year survival rates for PFS in the early pTNM stage were 97.6% (95% CI: 94.9–100.0%), 89.2% (95% CI: 83.8–94.9%) and 85.6% (95% CI: 79.5–92.2%), and for OS, these rates were 97.6% (95% CI: 94.9–100.0%), 89.2% (95% CI: 83.9–94.9%) and 86.7% (95% CI: 80.8–93.0%). In the advanced pTNM stage, the median survival time for PFS and OS was 26.93 and 35.63 months, respectively. The 1‐, 3‐ and 5‐year survival rates for PFS and OS in the advanced pTNM stage were 76.8% (95% CI: 67.2–87.8%), 36.1% (95% CI: 25.7–50.7%) and 32.3% (95% CI: 22.2–46.9%), and 81.5% (95% CI: 72.6–91.5%), 47.7% (95% CI: 36.7–61.8%) and 36.1% (95% CI: 25.9–50.4%), respectively. Patients in the advanced pTNM stage exhibited lower PFS (HR = 4.956, 95% CI: 2.797–8.781, *P* < 0.001) and OS (HR = 4.830, 95% CI: 2.741–8.511, *P* < 0.001) compared with those in the early pTNM stage (*Figure* *S2* *A,D*).

Within the early pTNM stage, the CD3+/CD4+ cell–myosteatosis was assessed in three groups: 14 patients in Group 1, 51 patients in Group 2 and 59 patients in Group 3. The 1‐, 3‐ and 5‐year survival rates for PFS and OS in Group 1 were 85.7% (95% CI: 69.2–100.0%) versus 85.7% (95% CI: 69.2–100.0%), 57.1% (95% CI: 36.3–89.9%) versus 57.1% (95% CI: 36.3–89.9%) and 57.1% (95% CI: 36.3–89.9%) versus 57.1% (95% CI: 36.3–89.9%). In Group 2, the 1‐, 3‐ and 5‐year survival rates for PFS and OS were 98.0% (95% CI: 94.3–100.0%), 89.6% (95% CI: 81.4–98.7%) and 80.5% (95% CI: 69.8–92.8%), and 98.0% (95% CI: 94.3–100.0%), 89.8% (95% CI: 81.7–98.7%) and 83.4% (95% CI: 73.5–94.6%), respectively. For Group 3, the 1‐, 3‐ and 5‐year survival rates for PFS and OS were 100.0% (95% CI: 100.0–100.0%) versus 100.0% (95% CI: 100.0–100.0%), 96.5% (95% CI: 91.8–100.0%) versus 96.5% (95% CI: 91.8–100.0%) and 96.5% (95% CI: 91.8–100.0%) versus 96.5% (95% CI: 91.8–100.0%). Patients in Group 1 exhibited shorter PFS (HR = 0.315, 95% CI: 0.165–0.598, *P* < 0.001) and OS (HR = 0.307, 95% CI: 0.159–0.590, *P* < 0.001) (*Figure* *S2* *B,E*).

In the advanced pTNM stage, there were 10 patients in Group 1, 36 patients in Group 2 and 20 patients in Group 3. The 1‐, 3‐ and 5‐year survival rates for PFS and OS in Group 1 were 60.0% (95% CI: 36.2–99.5%) versus 70.0% (95% CI: 46.7–100.0%), 10.0% (95% CI: 1.6–64.2%) versus 20.0% (95% CI: 5.8–69.1%) and 10.0% (95% CI: 1.6–64.2%) versus 10.0% (95% CI: 1.6–64.2%). For Group 2, the 1‐, 3‐ and 5‐year survival rates for PFS and OS were 71.3% (95% CI: 57.8–88.1%), 26.8% (95% CI: 15.0–47.8%) and 23.4% (95% CI: 12.4–44.3%), and 74.5% (95% CI: 61.4–90.4%), 40.6% (95% CI: 26.9–61.4%) and 25.0% (95% CI: 13.8–45.4%), respectively. In Group 3, the 1‐, 3‐ and 5‐year survival rates for PFS and OS were 95.0% (95% CI: 85.9–100.0%) versus 100.0% (95% CI: 100.0–100.0%), 67.0% (95% CI: 48.3–92.8%) versus 73.7% (95% CI: 56.3–96.4%) and 60.3% (95% CI: 41.0–88.7%) versus 68.4% (95% CI: 50.4–92.9%). Patients in Group 1 had worse PFS (HR = 0.400, 95% CI: 0.247–0.649, *P* < 0.001) and OS (HR = 0.414, 95% CI: 0.260–0.658, *P* < 0.001) (*Figure* *S2* *C,F*).

### Construction of nomograms to predict progression‐free survival and overall survival

**Figure 4:**
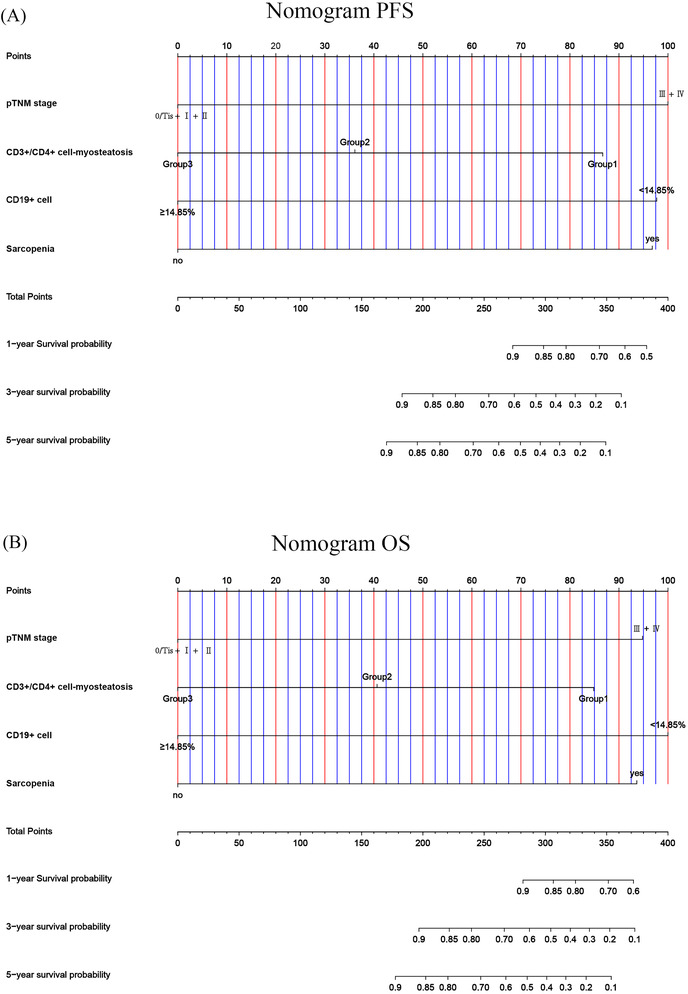
Nomogram for predicting the 1‐, 3‐ and 5‐year survival probability of (A) progression‐free survival (PFS) and (B) overall survival (OS). Group 1: CD3+/CD4+ cell ≥ 42.05% and myosteatosis; Group 2: CD3+/CD4+ cell ≥ 42.05% and without myosteatosis, or CD3+/CD4+ cell < 42.05% and without myosteatosis; and Group 3: CD3+/CD4+ cell < 42.05% and without myosteatosis. pTNM, pathological tumour–node–metastasis.

**Figure 5:**
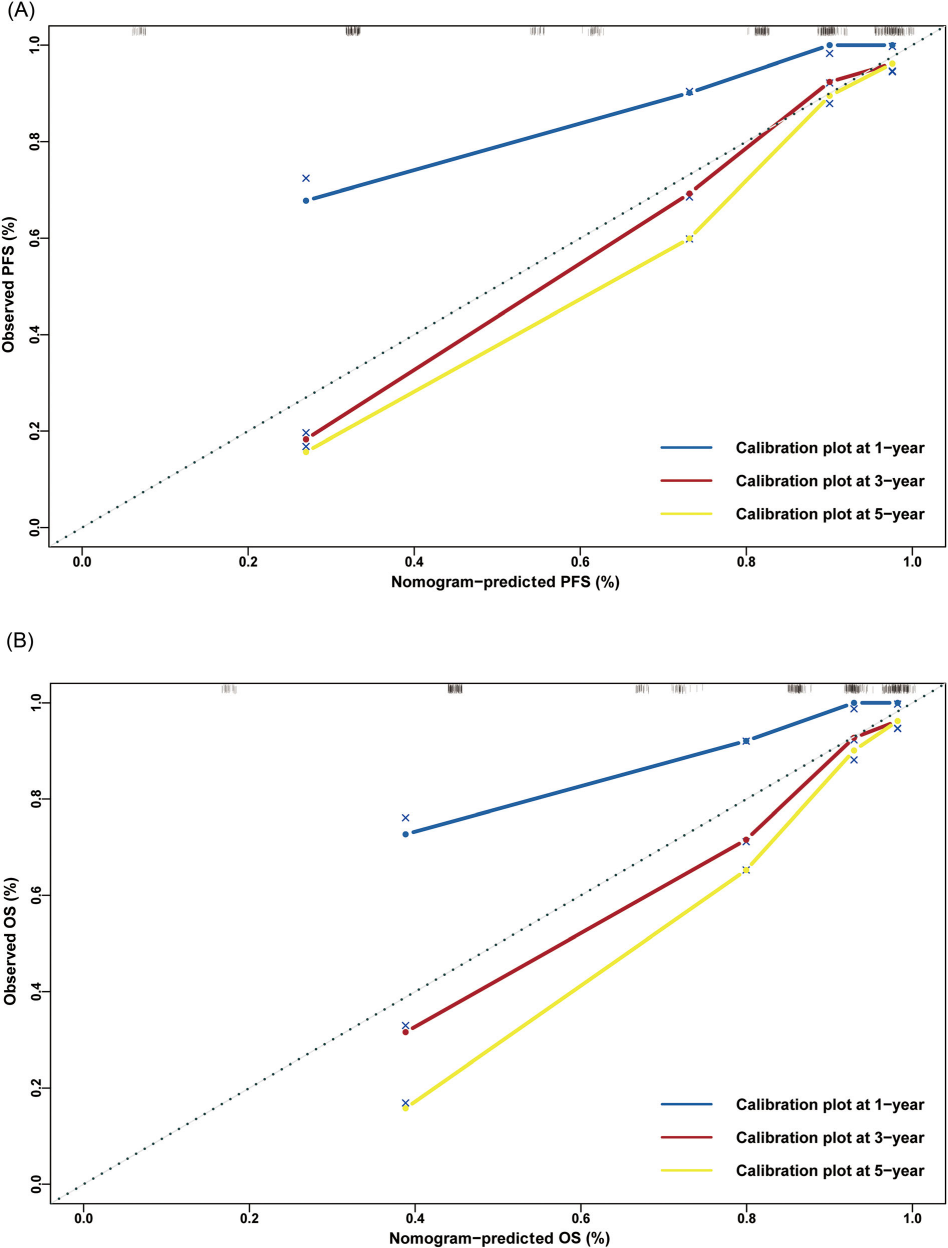
Calibration curves for predicting (A) progression‐free survival (PFS) and (B) overall survival (OS) at 1, 3 and 5 years.

This study determined that independent prognostic factors for PFS and OS were CD19+ cells, pTNM stage, sarcopenia and CD3+/CD4+ cell–myosteatosis. The C‐index and 95% CI for predicting the survival probability of PFS and OS were 0.839 (0.798–0.880) and 0.836 (0.792–0.879), respectively (*Figure* *4* *A,B*). The calibration analysis demonstrated that the nomograms accurately predicted the 3‐ and 5‐year survival rates of PFS and OS in patients (*Figure* *5* *A,B*).

## Discussion

Gastric cancer stands as a prevalent digestive tract malignancy in China, ranking third in both incidence and mortality rates.^21^ Despite advancements in the treatment modalities for gastric cancer patients, leading to improved survival rates, a substantial number still face unfavourable prognoses.^22^ Thus, there is an urgent imperative to identify novel indicators for accurate prognosis prediction among patients who underwent gastric cancer surgery. Inflammation, a well‐established cancer risk factor, significantly contributes to malignant tumour development and progression.^23^ Infiltration of tumours by inflammatory cells has emerged as an independent prognostic factor influencing patient outcomes.^24^ While previous studies have concentrated on immune cell infiltration within the tumour microenvironment,^25^ ^,^ ^S8^ the exploration of peripheral blood lymphocyte subsets in tumour patients remains relatively underexplored. The maintenance of a normal immune state crucially hinges on the coordination of diverse immune cells, particularly peripheral lymphocyte subpopulations. The constancy of lymphocyte subsets is perturbed in pathological states. Prior investigations have highlighted the favourable impact of higher peripheral blood levels of CD3+/CD8+ cells and lower levels of CD3−/CD56+ cells on OS in patients with nasopharyngeal carcinoma.^26^ A retrospective study involving 74 patients with advanced non‐small cell lung cancer during immunotherapy demonstrated prolonged OS with elevated levels of CD3+/CD4+ and CD8+ T cells and reduced levels of NK cells.^27^ In a more extensive retrospective study comprising 482 patients with metastatic breast cancer, high levels of CD3+ T cells and CD3+/CD4+ T cells were associated with a poorer prognosis.^28^ Notably, patients with elevated circulating NK cell counts in progressive gastric cancer exhibited a more favourable prognosis.^29^ Additionally, myosteatosis, a novel indicator of nutritional status, has demonstrated predictive capabilities for the prognosis of various solid tumours, including gastric cancer.^29^ ^,^ ^S9^

In our current study, Cox regression analysis of peripheral lymphoid subpopulations revealed an unfavourable prognosis for patients with elevated CD3+/CD4+ cell levels, a high CD4+/CD8+ ratio and diminished CD19+ cell counts. Examination of body composition unveiled that patients with sarcopenia and myosteatosis exhibited poorer prognoses. This study, for the first time, explored the association between CD3+/CD4+ cell–myosteatosis and the prognosis of patients who underwent surgery for gastric cancer. The findings established CD3+/CD4+ cell–myosteatosis as an independent prognostic factor for both PFS and OS in patients who underwent radical gastric cancer surgery. Additionally, pTNM stage, sarcopenia and CD19+ cell counts were identified as independent prognostic factors for PFS and OS.

Potential mechanisms underlying the predictive capabilities of CD3+/CD4+ cell binding myosteatosis in gastric cancer surgery outcomes were considered. CD3+/CD4+ cells, pivotal players in the immune system, assume crucial roles in recruiting, activating and regulating various aspects of the adaptive immune response.^30^ While CD4+ T lymphocyte subpopulations may not share identical roles, they collectively contribute to tumour immunity.^31^ The distribution of T lymphocyte subpopulations varies significantly at different tumour stages, indicating their potential role as determinants of tumour progression.^32^ T helper (Th)1 cells mediate anti‐tumour immune responses, with Th2 and regulatory T (Treg) cells exhibiting immunosuppressive properties. In early tumorigenesis, anti‐tumour effects mediated by CTL and Th1 predominate. However, as tumours progress, the increasing number and proportion of tumours promote the rapid growth and dominance of T lymphocytes. Tregs, among them, secrete inhibitory cytokines such as interleukin (IL)‐10 and IL‐35, exerting immunosuppressive effects by influencing Antigen‐presenting cell function and inducing CD8+ cell depletion, thereby contributing to tumour development and progression.^33^ ^,^ ^S10^ Myosteatosis is postulated to result from aging and low‐grade chronic inflammation leading to ectopic deposition of adipose tissue.^34^ ^,^ ^S11^ Elevated fat density may signify inflammatory changes, with serum inflammatory cell levels correlating with prognosis in various tumours. The muscle microenvironment, comprising neutrophils, monocytes and T lymphocytes, secretes mediators influencing repair and remodelling during skeletal muscle injury.^35^ Consequently, poor muscle mass, reflected by myosteatosis, may imply underlying impaired host immune defence, affecting the recurrence of metastasis in patients.

Furthermore, the immune system may engage in interactions with tissue wasting, consequently contributing to tumour recurrence and distant metastasis. Principally, inflammatory cells augment the systemic inflammatory response through the secretion of cytokines such as tumour necrosis factor alpha, IL‐1 and interferon gamma, thereby eliciting appetite suppression and diminishing food intake.^36^ ^,^ ^S12^ Within the tissues of individuals afflicted with malignant tumours, the predominant inflammatory infiltrating cells consist primarily of T lymphocytes.^37^ In instances where tumour patients exhibit cachexia, significant alterations occur in key signalling factors within the body, thereby further reducing the number of T lymphocytes, disrupting T cell metabolism and impeding differentiation.^38^ ^,^ ^S13^ ^,^ ^S14^ Consequently, this disruption in immune system functionality leads to a decrease in immune surveillance of the tumour, ultimately resulting in tumour progression and recurrence.^39^

Despite the significant findings, certain limitations must be acknowledged. This study constitutes a single‐centre retrospective analysis with a relatively modest sample size. The exclusive focus on gastric cancer patients who underwent surgery might limit the generalizability of the findings. Additionally, the determination of optimal cut‐off values for lymphatic subgroups heavily depends on ROC curves, with potential variations based on regional factors. To enhance the robustness and generalizability of our conclusions, multicentre prospective studies with larger sample sizes across diverse malignancies are warranted.

## Conclusions

In summary, our study establishes associations between peripheral lymphoid subsets, body components and clinical outcomes in patients who underwent gastric cancer surgery. The novel combination of CD3+/CD4+ cells with myosteatosis emerged as a highly effective indicator, offering heightened predictive efficacy for identifying recurrence and metastasis in post‐surgery gastric cancer patients. These findings provide valuable insights into the prognostic landscape of gastric cancer and lay the groundwork for future research avenues.

## Conflict of interest statement

The authors declare no competing financial interests.

## Supporting information


**Data S1.** Supporting Information.

**Figure S1.** Flow chart of patients' election in this study.

**Figure S2.** pTNM stage related survival curve for (A) PFS and (D) OS; CD3+/CD4+ cell myosteatosis related survival curves in pTNM stages Tis/0, I and II for (B) PFS and (E) OS; CD3+/CD4+ cell‐myosteatosis related survival curves in pTNM stages III and IV for (C) PFS and (F) OS. Group 1: CD3+/CD4+ cell ≥ 42.05% and myosteatosis; Group 2: CD3+/CD4+ cell ≥ 42.05% and without myosteatosis, or CD3+/CD4+ cell < 42.05% and without myosteatosis; Group 3: CD3+/CD4+ cell < 42.05% and without myosteatosis.

## Data Availability

The material supporting the conclusion of this article has been included within the article.
