# Supplementary material for: CD3+/CD4+ cells combined with myosteatosis predict the prognosis in patients who underwent gastric cancer surgery
Source: J Cachexia Sarcopenia Muscle. 2024 Jun 18;15(4):1587–600. doi: 10.1002/jcsm.13517 (PMC11294046; doi:10.1002/jcsm.13517)
Supplement: Supplementary file 1 — Data S1. Supporting Information. [file JCSM-15-1587-s002.docx]

Supplementary references:

[S1] Schmidt, MA, Förtsch, C, Schmidt, M, Rau, TT, Fietkau, R, Distel, LV. Circulating regulatory T cells of cancer patients receiving radiochemotherapy may be useful to individualize cancer treatment. RADIOTHER ONCOL. 2012-07-01; 104 (1): 131-8.

[S2] Sun, H, Wang, H, Pan, H, Zuo, Y, Zhao, R, Huang, R, Xue, Y, Song, H. CD19 (+) B Cell Combined with Prognostic Nutritional Index Predicts the Clinical Outcomes of Patients with Gastric Cancer Who Underwent Surgery. Cancers (Basel). 2023-04-28; 15 (9):

[S3] An, Z, Hsu, MA, Gicobi, JK, Xu, T, Harrington, SM, Zhang, H, Pavelko, KD, Hirdler, JB, Lohse, CM, Nabavizadeh, R, Pessoa, RR, Sharma, V, Thompson, RH, Leibovich, BC, Dong, H, Lucien, F. A Novel PD-L1 Antibody Promotes Antitumor Function of Peripheral Cytotoxic Lymphocytes after Radical Nephrectomy in Patients with Renal Cell Carcinoma. J IMMUNOL. 2023-06-15; 210 (12): 2029-2037.

[S4] Nemec, U, Heidinger, B, Sokas, C, Chu, L, Eisenberg, RL. Diagnosing Sarcopenia on Thoracic Computed Tomography: Quantitative Assessment of Skeletal Muscle Mass in Patients underwent Transcatheter Aortic Valve Replacement. ACAD RADIOL. 2017-09-01; 24 (9): 1154-1161.

[S5] Molfino, A, Laviano, A, Rossi Fanelli, F. Contribution of anorexia to tissue wasting in cachexia. CURR OPIN SUPPORT PA. 2010-12-01; 4 (4): 249-53.

[S6] Laviano, A, Meguid, MM, Guijarro, A, Muscaritoli, M, Cascino, A, Preziosa, I, Molfino, A, Rossi Fanelli, F. Antimyopathic effects of carnitine and nicotine. CURR OPIN CLIN NUTR. 2006-07-01; 9 (4): 442-8.

[S7] Zitvogel, L, Pietrocola, F, Kroemer, G. Nutrition, inflammation and cancer. NAT IMMUNOL. 2017-07-19; 18 (8): 843-850.

[S8] Shamamian, P, Schwartz, JD, Pocock, BJ, Monea, S, Whiting, D, Marcus, SG, Mignatti, P. Activation of progelatinase A (MMP-2) by neutrophil elastase, cathepsin G, and proteinase-3: a role for inflammatory cells in tumor invasion and angiogenesis. J CELL PHYSIOL. 2001-11-01; 189 (2): 197-206.

[S9] Chen, BB, Liang, PC, Shih, TT, Liu, TH, Shen, YC, Lu, LC, Lin, ZZ, Hsu, C, Hsu, CH, Cheng, AL, Shao, YY. Sarcopenia and myosteatosis are associated with survival in patients receiving immunotherapy for advanced hepatocellular carcinoma. EUR RADIOL. 2023-01-01; 33 (1): 512-522.

[S10] van der Veeken, J, Gonzalez, AJ, Cho, H, Arvey, A, Hemmers, S, Leslie, CS, Rudensky, AY. Memory of Inflammation in Regulatory T Cells. CELL. 2016-08-11; 166 (4): 977-990.

[S11] Franceschi, C, Garagnani, P, Parini, P, Giuliani, C, Santoro, A. Inflammaging: a new immune-metabolic viewpoint for age-related diseases. NAT REV ENDOCRINOL. 2018-10-01; 14 (10): 576-590.

[S12] Fearon, KC, Glass, DJ, Guttridge, DC. Cancer cachexia: mediators, signaling, and metabolic pathways. CELL METAB. 2012-08-08; 16 (2): 153-66.

[S13] Rathmell, JC, Vander Heiden, MG, Harris, MH, Frauwirth, KA, Thompson, CB. In the absence of extrinsic signals, nutrient utilization by lymphocytes is insufficient to maintain either cell size or viability. MOL CELL. 2000-09-01; 6 (3): 683-92.

[S14] Palmer, CS, Ostrowski, M, Balderson, B, Christian, N, Crowe, SM. Glucose metabolism regulates T cell activation, differentiation, and functions. Front Immunol. 2015-01-01; 6 1.

Supplementary images:

Figure S1. Flow chart of patients’ election in this study.

Figure S2. pTNM stage related survival curve for (A) PFS and (D) OS; CD3+/CD4+ cell myosteatosis related survival curves in pTNM stages Tis/0, Ⅰ and Ⅱ for (B) PFS and (E) OS; CD3+/CD4+ cell-myosteatosis related survival curves in pTNM stages Ⅲ and Ⅳ for (C) PFS and (F) OS. Group 1: CD3+/CD4+ cell ≥ 42.05% and myosteatosis; Group 2: CD3+/CD4+ cell ≥ 42.05% and without myosteatosis, or CD3+/CD4+ cell < 42.05% and without myosteatosis; Group 3: CD3+/CD4+ cell < 42.05% and without myosteatosis.


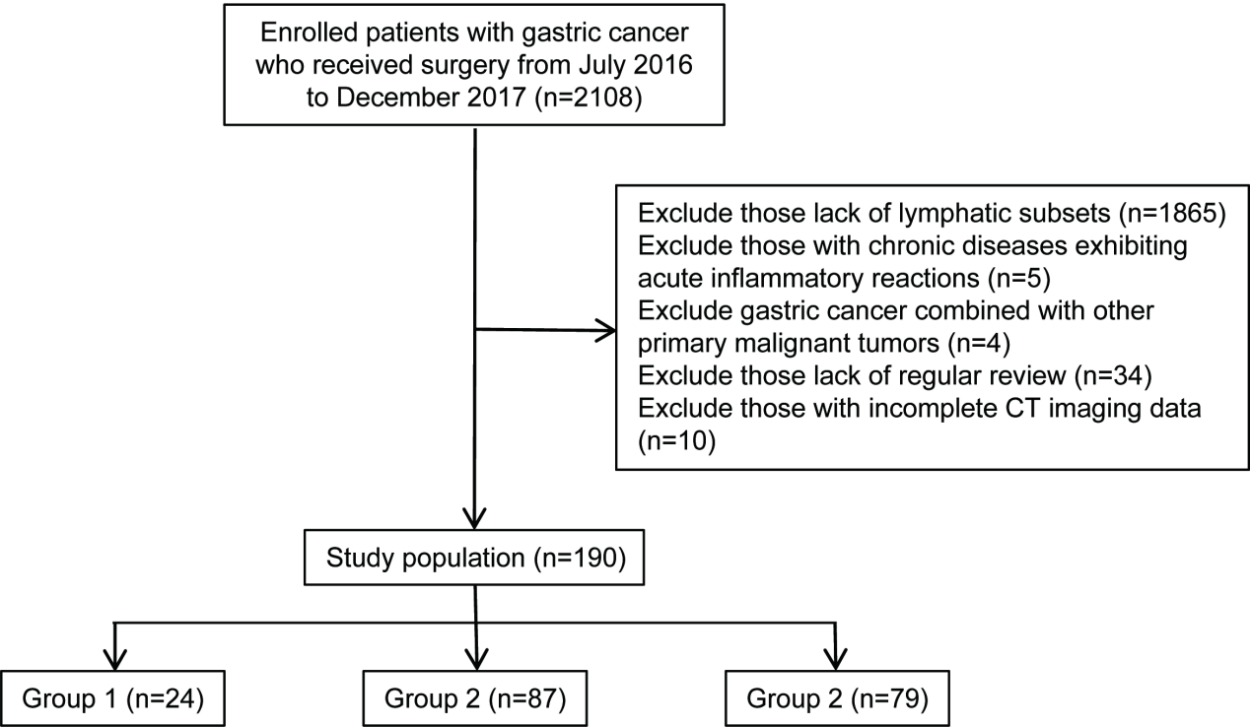


Figure S1. Flow chart of patients’ election in this study.


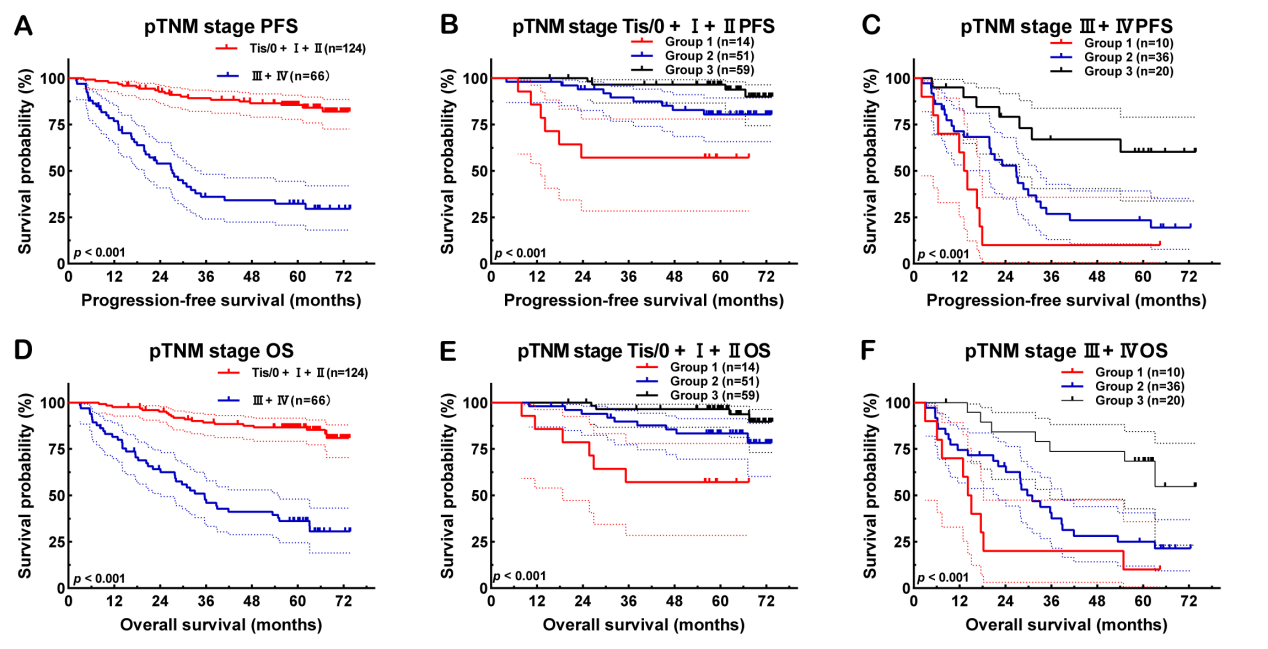


Figure S2. pTNM stage related survival curve for PFS (A) and OS (D); CD3+/CD4+ cell-myosteatosis related survival curves in pTNM stages Tis/0, Ⅰ and Ⅱ for PFS (B) and OS (E); CD3+/CD4+ cell-myosteatosis related survival curves in pTNM stages Ⅲ and Ⅳ for PFS (C) and OS (F).

Group 1: CD3+/CD4+ cell ≥ 42.05% and myosteatosis; Group 2: CD3+/CD4+ cell ≥ 42.05% and without myosteatosis, or CD3+/CD4+ cell < 42.05% and without myosteatosis; Group 3: CD3+/CD4+ cell < 42.05% and without myosteatosis.
