# Supplementary figures and images for: CD3+/CD4+ cells combined with myosteatosis predict the prognosis in patients who underwent gastric cancer surgery
Source: J Cachexia Sarcopenia Muscle. 2024 Jun 18;15(4):1587–600. doi: 10.1002/jcsm.13517 (PMC11294046; doi:10.1002/jcsm.13517)

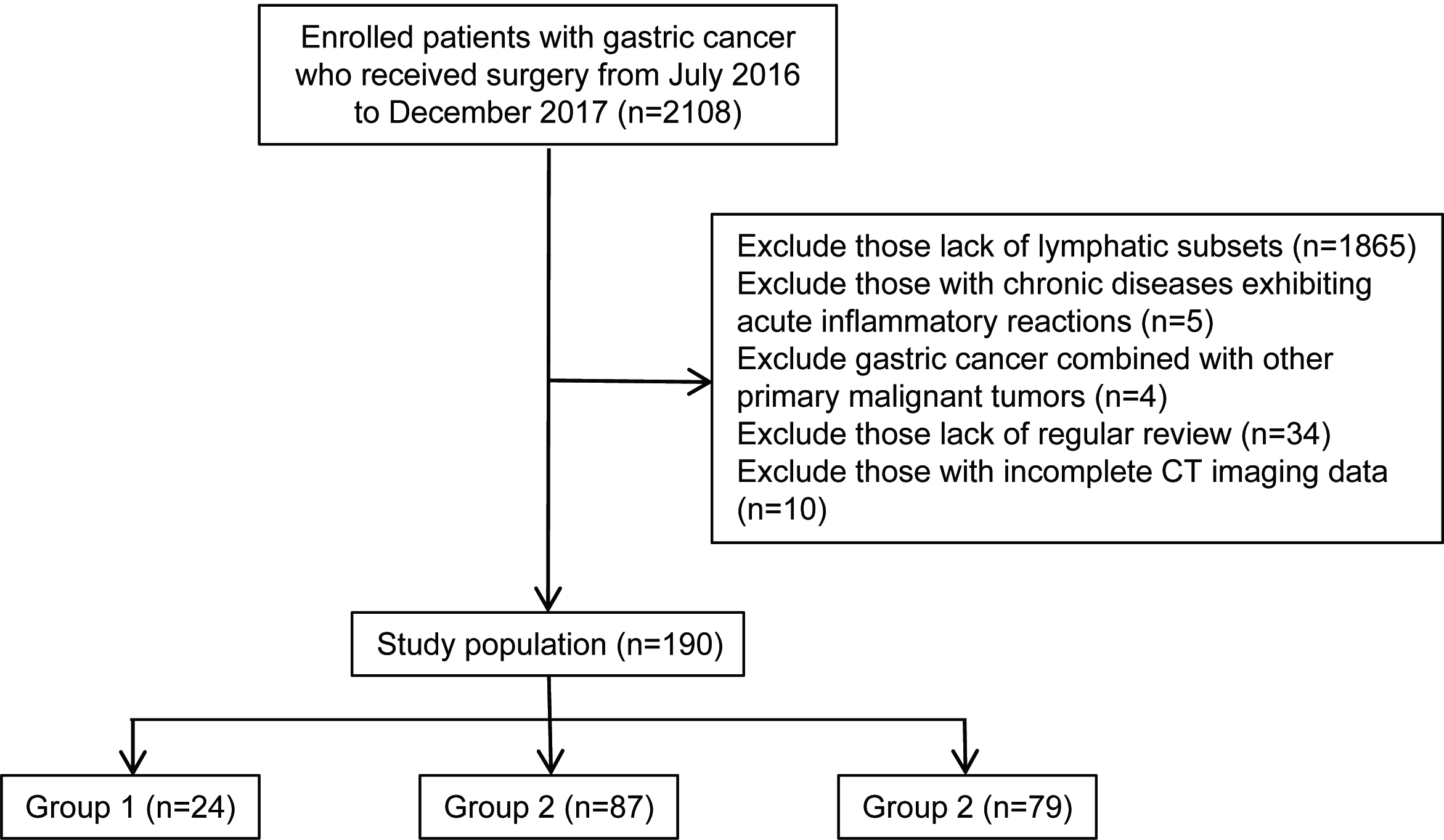

Supplement: Supplementary file 2 — Figure S1. Flow chart of patients' election in this study. [file JCSM-15-1587-s003.tif]

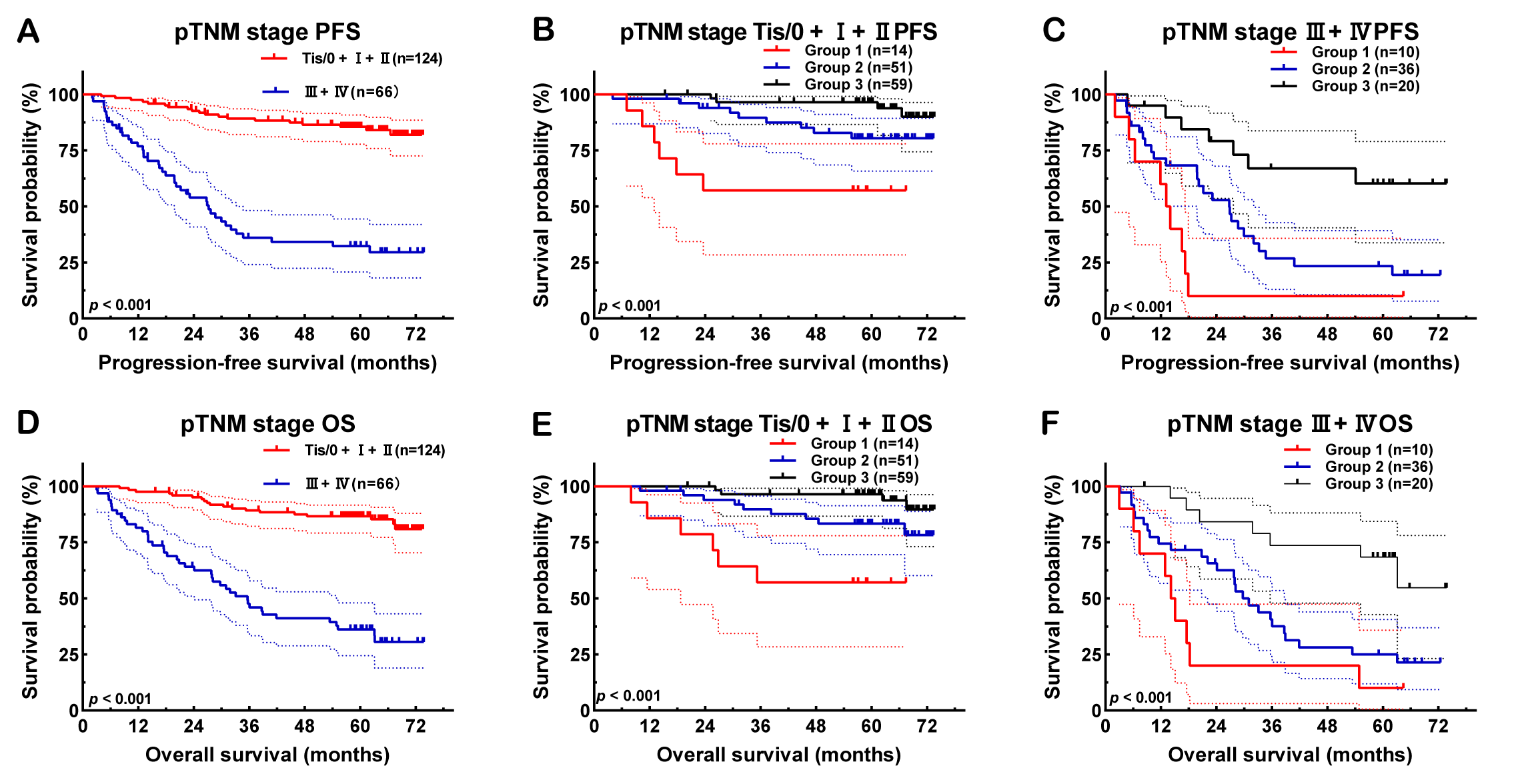

Supplement: Supplementary file 3 — Figure S2. pTNM stage related survival curve for (A) PFS and (D) OS; CD3+/CD4+ cell myosteatosis related survival curves in pTNM stages Tis/0, I and II for (B) PFS and (E) OS; CD3+/CD4+ cell‐myosteatosis related survival curves in pTNM stages III and IV for (C) PFS and (F) OS. Group 1: CD3+/CD4+ cell ≥ 42.05% and myosteatosis; Group 2: CD3+/CD4+ cell ≥ 42.05% and without myosteatosis, or CD3+/CD4+ cell < 42.05% and without myosteatosis; Group 3: CD3+/CD4+ cell < 42.05% and without myosteatosis. [file JCSM-15-1587-s001.tif]
